# Comparison of the patient radiation exposure during coronary angiography and angioplasty procedures using trans-radial and trans-femoral access

**DOI:** 10.15171/jcvtr.2016.15

**Published:** 2016-06-28

**Authors:** Ali Tarighatnia, Amir Hossein Mohammad Alian, Morteza Ghojazadeh, Alir Reza Farajollahi

**Affiliations:** ^1^Immunology Research Center, Tabriz University of Medical Sciences, Tabriz, Iran; ^2^Department of Medical Physics, Tabriz University of Medical Sciences, Tabriz, Iran; ^3^Interventional Cardiology Unit, Aalinasab Hospital, Tabriz, Iran; ^4^Liver And Gastrointestinal Disease Research Center, Tabriz University of Medical Sciences, Tabriz, Iran; ^5^Medical Education Research Centre, Tabriz University of Medical Sciences, Tabriz, Iran

**Keywords:** Tran Radial Access (TRA), Trans Femoral Access (TFA), Patient Radiation Dose

## Abstract

***Introduction:*** Cardiac catheterization procedure through the trans-radial access (TRA) have shown many clinical advantages over the trans-femoral (TFA), but despite its advantages, there are serious concerns regarding higher possible radiation dose for the patients and operators in TRA. This study was planned to compare the patients’ radiation dose associated with TRA and TFA during coronary angiography (CA) and percutaneous transluminal coronary angioplasty (PTCA).

***Methods:*** Of 700 candidates for angiography, 326 patients were entered the study. All the procedures were carried out by one interventional cardiologist employing the same angiography unit in Aalinasab hospital and the patients’ dose area product (DAP), air kerma (AK), fluoroscopy time (FT) and cine film time (CFT) were then determined in both access groups (TRA,TFA) in CA, PTCA and CA+PTCA procedures.

***Results:*** The mean FT, CFT and AK values in both TRA & TFA groups were the same in all procedures (*P*>0.05). The mean DAP in CA+PTCA procedures was 6704.01±3243.23 µGym^2^ in femoral access compare with 5647.46±2797.74 µGym^2^ in radial access, which were significantly less than that in TFA with *P*= 0.02.

***Conclusion:*** On the basis of the results obtained in this study, no differences were found in patients’ radiation dose in both access groups, therefore with regard to comparatively more clinical advantages associated with the Trans-radial access technique it might be a good substitute for Trans-femoral access.

## Introduction

With the ever-increasing rate of cardiovascular diseases, especially coronary artery diseases (CAD), the need for angiography and angioplasty of coronary arteries (coronary angiography [CA] and percutaneous transluminal coronary angioplasty [PTCA]) has increased. It is expected that the total number of these procedures will increase several folds by 2020 with the development of new angiography systems and with an increase in skills necessary for carrying out complex coronary artery treatment procedures.^1,2^

Since angiography and angioplasty procedures are safe and highly efficient for the diagnosis and treatment of CAD, they can have a great role in decreasing patient mortality rate^3^; however, their most important disadvantage is the high patient and consequently personnel radiation dose.^4^

In such procedures the highest patient radiation dose is due to the direct exposure to the thorax and in cases in which there is a need for the continuation of treatment or repetition of the procedure, the patient receives a higher dose compared to other imaging techniques, with increased potential for biologic effects, including skin injuries and the possible radiation-induced injuries such as cancer.^5-7^

One of the parameters affecting the patient radiation dose and the clinical consequence is the type of vascular access which has attracted the attention of many researchers.^8,9^ The results of clinical studies have shown that trans-radial access (TRA) is superior to trans-femoral access (TFA) due to a reduction in mortality in patients undergoing Primary PCI for STEMI,^10,11^ decreased access site complications,^9,10,12^ high safety and efficacy, a decrease in the duration of hospitalization, rapid recovery and return to daily routines, and a decrease in costs.^13-15^ Some of studies indicated higher patient radiation dose associated by TRA^16-18^ and some other related to the lower and equal patient dose,^19-21^ therefore there is controversy over the amount of radiation dose that received by the patient in the TRA in comparison with the TFA and there is no consensus over it.^16,22^ On the other hand, many of studies in this respect have not been appropriate and comprehensive and have only evaluated the absorbed radiation dose as a secondary aim of the study along with clinical evaluations.

The aim of present study is to evaluate radiation dose associated with the patient when using radial access technique and compare it when femoral access is applied in CA and PTCA procedures.

## Materials and Methods

This randomized study was carried out for 9 months from September 2013 to June 2014 in the Department of Angiography, Aalinasab hospital, Tabriz, Iran, which is a hospital in the north-west of Iran with a high referral rate**.** In this hospital, cardiac catheterization procedures are carried out using TRA and TFA techniques.

The angiography team in the hospital consists of cardiologist, nurses and radiologic technologists who are responsible for adjusting and delivering radiation doses and also for supervision of radiation protection procedures. In this study, from 700 patients who were candidates for CA and angioplasty, 326 patients were entered the study. Patients with a negative Allen test, a history of coronary bypass procedure, valvular heart disease and a history of unsuccessful procedure and also emergency cases were excluded from the study. The patients were randomly divided into TRA and TFA groups using the RandList 1.2 software program. Stratified sampling technique was used to classify the procedure in 3 groups of CA, PTCA and CA + PTCA. The patients were placed in one of these groups based on their clinical needs and the operator’s decision, all of which are separately presented in Table 1.

**Table 1:** Comparison of radiation factors in different coronary procedures with TRA and TFA techniques

**Procedure**	**CA**			**PTCA**			**CA+PTCA**		
	**TRA**	**TFA**	**P**	**TRA**	**TFA**	**P**	**TRA**	**TFA**	**P**
Number	37(22.6%)	37(22.6%)		74 (45.39%)	74 (45.39%)		52(31.9%)	52(31.9%)	**-**
DAP (µGym^2^)	1732.55 (625.9-3656)	1949.71 (679.7-10781)	0.17	4343.88 (521.8-11843.1)	5277.03 (480.9-19452)	0.09	5647.46 (1782-13614)	6740 (1752.9-18609.9)	0.001
AK (mGy)	233.88 (74.7-526)	210.78 (86.6-433)	0.9	734.36 (85.9-2336.1)	854.51 (93-3464)	0.1	891.36 (251.3-2324)	1041.281 (301-2545)	0.07
FT (min)	3.33 (0.78-9.5)	1.77 (0.5-8.36)	0.9	8.4 (1-21.1)	8.76 (0.9-37.05)	0.38	11.24 (3.5-25.7)	10.78 (2.41-42.3)	0.63
CFT (s)	25.27 (17-39)	24.4 (16-35)	0.78	33.77 (9-71)	37.65 (9-91)	0.13	57.76 (27-1.5)	61.31 (24-115)	0.2

Data are shown with Mean (min-max) and N (%).

All the procedures were carried out by one operator because factors such as the operator’s skill and experience can affect the patient radiation exposure.^4,8^ The operator in the present study was an interventional cardiologist who has vast experience in carrying out angiography and angioplasty with both radial and femoral access. In addition, only a Siemens angiography unit (Model Axiom Artis dfc, Germany) was used to eliminate the effect of influencing factors such the system characteristics. This angiography unit has the variable pulse and frame rate and has 3 magnification modes. These variables were the same with both techniques evaluated in the present study.

Before the study, the accuracy and reproducibility of exposure factors (time and kVp) were evaluated with the use of Dia dose and Dia volt quality control kits (PTW-Freiburg, Germany) at the reference point (at a distance of 15 cm over the isocenter point).

During each procedure, apart from of dosimetry factors, such as air kerma (AK),dose area product (DAP), fluoroscopy time (FT) and cine film time/cine acquisition (CFT), other demographic and clinical data of patients were recorded in both TFA and TRA techniques.

### Analysis of data

Data were analyzed with descriptive statistical methods (Mean ± SD and frequency [%]), the mean comparison test, multi-factor analysis of variance (ANOVA) and chi-square test using SPSS 17. The *P *< 0.05 was considered significant.

## Results

The patients’ demographic information along with their clinical status are shown in the Table 2. From the table, the means of age, height, weight, body mass index (BMI) and clinical status of patients also their gender were the same in both TRA and TFA techniques, with no significant differences (*P* > 0.05).

**Table 2:** Comparison of some demographic and clinical characteristics of patients in both TRA and TFA techniques

**Parametric data**	**Access**		
	**TRA (n=163)**	**TFA (n=163)**	**P value**
Age (y)	59.55±10.3	60.65±11.2	0.36
Weight (kg)	76.14±12.15	76.57±11.96	0.74
Height (cm)	166.54±8.94	165.77±9.69	0.46
Male	119 (73%)	114 (70%)	0.53
Female	44 (27%)	49 (30%)	
BMI (kg/m^2^)	27.54±4.35	27.88±4.04	0.46
Clinical status			
Unstable angina	83 (51%)	80 (49%)	0.78
Stable angina	80 (49%)	83 (51%)	

Data are shown with mean ± SD and n (%).

Table 1 presents the patients’ radiation doses with the TRA and TFA techniques separately for each procedure in terms of FT, CFT, DAP and AK parameters. As shown in the table, the patients’ radiation doses were the same in both TRA and TFA techniques in terms of FT, CFT and AK parameters in all the three CA, PTCA and CA + PTCA procedures, without any significant differences (P>0.05). Furthermore, based on the results, although the patients’ DAP was not significantly different between CA and PTCA procedures, this factor was 6704.01± 3243.23 (1752-18609.9 µGym^2^) and 5647.46±2797.74 (1782-13614 µGym^2^) in TFA and TRA respectively, indicating that the DAP in the patients who underwent angioplasty following angiography procedures (CA + PTCA) through the TFA was 1056.55 µGym^2^ higher than that of trans radial access.

Statistical analysis has shown that the differences in mean DAP between the two access group is significant with *P *< 0.001.

Since the mean absorbed radiation dose of patients in terms of the above-mentioned parameters in coronary procedures can affect the cardiologist’s decision on selecting the type of access, the mean absorbed radiation doses of the patients in the two groups were compared without considering the type of the procedure, the results of which are presented in Table 3. As the results show, despite the fact that the skin doses (AK) and the FT were the same, the mean DAP values of the patients were higher in TFA in comparison to TRA technique with *P *< 0.05.

**Table 3:** Comparison of the mean absorbed radiation doses of patients with the TRA and TFA techniques

**Patient’ dose**	**Access**		
	**TRA**	**TFA**	***P*** **value**
AK (mGy)	619.85±40.44	702.19±35.87	0.12
FT (min)	7.66±0.46	7.1±0.41	0.36
CFT (s)	38.93±1.39	41.12±1.23	0.24
DAP (µGym^2^)	3907.96±249.7	4643.58±221.4	0.02

Data are shown with mean± SD

## Discussion

Studies have shown that the TRA technique to have more clinical advantages over the TFA technique for cardiac catheterization procedures.^9,23^ However, despite its clear advantages, the clinicians have not generally preferred to carry out CA and PTCA procedures extensively due to the higher chance of the radiation dose for the patients and the operators in radial in comparison to femoral access,^24^ even though there is no general consensus on the higher exposure rate generally associated with TRA. For instance, a number of studies have compared the patient radiation dose, essentially base on the mean FT values,^25,26^ while some other have primarily evaluated clinical parameters and peripherally investigated FT mean values.^22,27,28^

However, the results of the present study did not show any significant differences in the mean FT values between the TRA and TFA techniques in different procedures (*P *> 0.05), which are consistent with Yiğit et al^22^ on CA procedures, and contradict the findings of other study.^27^

The results of this study showed that the mean FT with the TRA technique was significantly higher than that with the TFA technique in the CA procedure (*P *< 0.05). The discrepancies between the results of studies in relation to CA procedures might be predominantly attributed to the longer FT in the TRA technique in order to visualize the catheter path and guide wire under fluoroscopy until they reach the coronary ostium.

In addition, based on the results of this study, there was no significant difference in the mean FT in angioplasty procedures between the both access techniques, which are consistent with the results of a study by Lehmann et al^28^ and different from those of studies by Rao et al and Suleiman et al.^25,26^ In these two studies the mean FT was reported to be longer in the TRA compared to the TFA technique (*P *< 0.05). The discrepancy between the results of studies might be attributed to differences in the angiographic characteristics of the vessels in question and differences in the operators’ experience.

Comparison of FT in the sequential examination of CA + PTCA showed that none of the access techniques was superior to the other, which inconsistent with the results of studies by Geijer and Persliden^20^ and Sandborg et al^29^; however, studies by Larrazet et al^30^ and Shah et al^18^ showed that FT was 29% and 38% longer, respectively, with the TRA technique compared to the TFA technique. The differences between the results of the present study and those of Larrazet et al^30^ and Shah et al might be attributed to the number of lesions treated with angioplasty and the difference in experience levels of operators carrying out the procedure. Thus there is a direct correlation between FT and operator experience as reported by Ball et al.^31^

Since the overall patient radiation dose in CA and PTCA procedures is the total dose received during fluoroscopy and cine film and that the FT has a minor role in the overall patient radiation dose compared to CFT, therefore FT itself cannot be considered an appropriate criterion for comparison of patient radiation doses in different access techniques because it does not provide a reliable risk estimation for the radiation biological hazards,^9,24^ thus, in this study, patients’ DAP, which is an indication of the overall radiation dose they have received during angiography and angioplasty procedures,^21^ was evaluated and compared in both access techniques.

The results of the present study did not show significant differences in the mean patients’ DAP between the two access techniques in the CA procedure (P>0.05), which is in agreement with the results of some similar studies^20,21^ and different from those of some others.^16,18^ Since there is a linear relationship between BMI and DAP,^32^ the differences between the results might dueto the patients higher BMI in the TRA group in comparison with TFA in the study by Neill et al.^32^ In this study there was no significant differences in BMI in two access group with *P *> 0.05.

In addition, a higher radiation dose that found in a study by Vlachadis et al with the radial access might be related to the operators’ lower experience, because the femoral access was routine technique of angiography in the centre that study was conducted.^16^

Moreover based on the results of this study neither of the access groups have shown superiority over the other in mean DAP values in PTCA, which is agreement with the results of studies by Kuipers et al^21^ and Geijer et al,^20^ but does not corresponds to the result of the study by Brasselet et al^33^ and Larrazet et al^30^ who reported a lower patient DAP in angioplasty through the femoral artery compared to that through the radial artery, such a difference might be resulted from differences in the number of coronary lesions and the lower mean BMI of patients in the TRA compared to the TFA group.

Furthermore, the finding of the present study indicated 16% decrease in DAP in the TRA technique compared to the TFA in the sequential tests of CA + PTCA, which corresponded to the results by Gray et al^34^ and Neill et al^32^ but contraindicated the findings of the studies evaluating patient’ DAP higher in trans radial access^27,33^; This discrepancy could have resulted from further collimation of the radiation field in the TRA in this study.

The results of this study have also shown that the mean patients DAP values in TRA, irrespective of the type of the procedures significantly lower than that of TFA, which is not coincide with results of studies carried out by Rigattieri et al^35^ and Brueck et al^27^ such discrepancy in the results might be attributed to the use of more collimated radiation beam size in TRA than the TFA technique in this study.

Since some cases of skin injuries have been reported after angiography and angioplasty procedures due to high radiation dose in these procedures,^6^ it is important to estimate the skin dose in such procedures . Therefore, in addition to DAP and FT, the patients’ AK was also evaluated in the present study because unlike to DAP the size of the field does not affect it due to measurement of the point dose,^4^ providing a better estimate of skin injurie.^4,17^

The results of the present study showed no significant differences in patient absorbed radiation doses in terms of AK between the TRA and TFA in different procedures (*P*=0.12), which is consistent with the results of studies by Jolly et al^4^ and Michael et al^36^ and contrary to the results of a study by Mercuri et al.^17^ Mercuri et al showed higher patient AK in the TRA groups compared to the TFA group; the discrepancy between the two studies could be explained by differences in the imaging systems used, the operator’ skill and the characteristics of angiography vessels.

## Conclusion

On the basis of the results, obtained in this study, no differences were found in patient’s radiation dose in both access groups, therefore with regard to comparatively more clinical advantages associated with the TRA technique it might be a good substitute for TFA.

## Ethical approval

All the patients signed informed consent forms before the CA and PTCA procedures. The study protocol was approved by the Ethics Committee of Tabriz University of Medical Sciences under the code TBZ R355.

## Competing interests

Authors declare no conflict of interests in this study.
